# O-Glycosylation of NnTreg Lymphocytes Recognized by the *Amaranthus leucocarpus* Lectin

**DOI:** 10.1155/2013/506807

**Published:** 2013-09-24

**Authors:** María C. Jiménez-Martínez, Ricardo Lascurain, Aniela Méndez-Reguera, Sergio Estrada-Parra, Iris Estrada-García, Patricia Gorocica, Salvador Martínez-Cairo, Edgar Zenteno, Raúl Chávez

**Affiliations:** ^1^Unidad de Investigación, Instituto de Oftalmología Fundación Conde de Valenciana, 06800 México, DF, Mexico; ^2^Laboratorio de Inmunología, Departamento de Bioquímica, Facultad de Medicina, (UNAM), P.O. Box 70159, 04510 México, DF, Mexico; ^3^Departamento de Inmunología, Escuela Nacional de Ciencias Biológicas, IPN, 11340 México, DF, Mexico; ^4^Departamento de Bioquímica, Instituto Nacional de Enfermedades Respiratorias, 4502 México, DF, Mexico

## Abstract

O-glycosidically-linked glycans have been involved in development, maturation, homing, and immune regulation in T cells. Previous reports indicate that *Amaranthus leucocarpus* lectin *(ALL)*, specific for glycans containing galactose-N-acetylgalactosamine and N-acetylgalactosamine, recognizes human naïve CD27^+^CD25^+^CD4^+^ T cells. Our aim was to evaluate the phenotype of CD4^+^ T cells recognized by *ALL* in peripheral blood mononuclear cells obtained from healthy volunteers. CD4^+^ T cells were isolated by negative selection using magnetic beads-labeled monoclonal antibodies; the expression of T regulatory cell phenotypic markers was assessed on *ALL*-recognized cells. In addition, IL-4, IL-10, IFN-*γ*, and TGF-*β* intracellular production in *ALL* ^+^ cells was also evaluated. The analyses of phenotypic markers and intracellular cytokines were performed through flow cytometry. *ALL*-recognized CD4^+^ T cells were mainly CD45RA^+^, CCR7^+^ cells. Although 52 ± 10% CD25^+^Foxp3^+^ cells were positive to *ALL*, only 34 ± 4% of *ALL* ^+^ cells corresponded to CD25^+^Foxp3^−^ cells. Intracellular cytokines in freshly obtained *ALL* ^+^CD4^+^ T cells exhibited 8% of IL-4, 15% of IL-10, 2% of IFN-*γ*, and 15% of TGF-*β*, whereas *ALL* ^−^CD4^+^ T cells depicted 1% of IL-4, 2% of IL-10, <1% of IFN-*γ*, and 6% of TGF-*β*. Our results show that galactose-N-acetylgalactosamine and N-galactosamine-bearing CD4^+^ T cells expressed phenotypic markers of NnTreg cells.

## 1. Introduction

Lectins specific for O-glycosidically-linked glycans, containing galactose-N-acetylgalactosamine or N-acetylgalactosamine, have been used to study T-cell activity, as reviewed by [1, 2]. In these studies, the lectin from *Artocarpus heterophyllus* is considered to be mitogenic for human CD4^+^ T cells [3], whereas *Artocarpus lakoocha* has antiproliferative effect on human leukemic cells [4], and *Dolichos biflorus* agglutinin is used to isolate leukemic T cells [5].

*Amaranthus leucocarpus lectin* (*ALL*) is a nonmitogenic lectin with specificity for galactose-N-acetylgalactosamine (Gal*β*1,3 GalNAc*α*1,O-Ser/Thr) or N-acetylgalactosamine (GalNAc*α*1,O-Ser/Thr) [6]. In contrast to other GalNAc-specific lectins such as *S. sclarea* [7] or PNA [8], the recognition of *ALL* is limited when GalNAc residues are arranged in clusters, as demonstrated by using glycopeptides with different GalNAc distribution [9, 10]. This lectin binds murine medullary thymocytes [11] and a human peripheral blood CD4^+^ T cell subset with phenotypic markers CD25, CD27, and CD45RA [12]. Interestingly, *ALL* is capable of inducing suppression of the immune response in mice [13], and it recognizes dexamethasone-resistant thymocytes with increased GalNAc transferase-activity [14]. It has been shown that dexamethasone administration to mice allows the survival of functional CD4^+^CD25^+^ T regulatory cells in the thymus [15]. Likewise, the dexamethasone treatment in asthma patients promotes differentiation toward T regulatory cells by a Foxp3-dependent mechanism [16], and when patients, receiving allogeneic lymphocyte transplantation, are treated with glucocorticoid, graft versus host disease is suppressed by expansion of CD4^+^CD25^+^Foxp3^+^ T cells [17].

The existence of T-cell subsets with regulatory capacity of the immune response, expressing CD4, CD25, and Foxp3 has been evidenced [18]. Naturally occurring CD4^+^CD25^+^ regulatory T cells (nTregs) represent a major lymphocyte population engaged in the maintenance of immune tolerance as reviewed in [18]. CD4^+^CD25^+^ nTregs are differentiated in the normal thymus as a functionally distinct subpopulation of T cells [19, 20]. In humans, the CD4^+^CD25^+^ nTregs are CD27^+^CCR7^+^Foxp3^+^, and most of these cells express CD45RO [21]. On the other hand, the existence of CD45RA^+^ Tregs that resemble a naïve cell subset (NnTreg) have been described [22, 23]. Glycosylation changes have also been reported in nTregs, suggesting that sialylation could be a regulatory ligand in CD4^+^CD25^+^ Foxp3^+^ cells [24]. In this context, O-glycosylation has been proposed to play a direct and powerful role in regulating T-cell function [14, 25, 26]. Recently, *ALL* has also shown a costimulatory effect on human CD4^+^ T cell activated via CD3 [27] turning *ALL* into a new tool to study O-glycans-bearing glycoproteins in T-cell populations. Thus, the aim of this work was to know whether the O-glycosidically linked structures recognized by *ALL* are expressed by a Treg subset. 

## 2. Material and Methods

### 2.1. Antibodies and Reagents

Phycoerythrin (PE)-labeled mouse IgG monoclonal antibodies (mAbs) against human IL-4, IL-10, and CTLA-4 and fluorescein isothiocyanate (FITC)-labeled antibodies against human IFN-*γ*, CD62L, Foxp3, and CyChrome-streptavidin were purchased from BD PharMingen (San Jose, CA, USA). FITC- and PE-labeled mouse anti-human CD45RA and CD45RO mAbs and anti-IgG-FITC were obtained from Southern Biotech, Inc. (Birmingham, AL, USA). FITC- and PE-labeled mAbs antihuman CCR7 and anti-hLAP (TGF-*β*1) were from RD Systems (Minneapolis, MN, USA). CD4 T-cell isolation Kit II (MiniMACS system) was obtained from Miltenyi Biotec (Auburn, CA, USA). Lymphoprep (Ficoll 1.077 density) was from Nycomed Pharma (Nyegaard, Oslo, Norway). RPMI-1640 culture medium, concanavalin A, FITC- and PE-labeled streptavidin, N-Acetyl-D-galactosamine (GalNAc), and salts were from Sigma Chemical Co. (St. Louis, MO, USA). Biotin was obtained from Pierce Chem, Co (Rockford, IL, USA). Sodium pyruvate, L-glutamine, and 2-mercaptoethanol were purchased from Gibco BRL. (Rockville, MD, USA). Fetal calf serum was from HyClone Labs. (Logan, UT, USA), and BD Cytofix/Cytoperm Kit and FASTImmune Kit (IFN-*γ*/IL-4) were from e-Biosciences (San Diego, CA, USA).

### 2.2. Lectin

*Amaranthus leucocarpus* seeds were obtained from Tulyehualco, Mexico, and the lectin (*ALL*) was purified by affinity chromatography [6] and subsequently labeled with biotin at a biotin/protein ratio of 2 : 1 [28].

### 2.3. Isolation of Peripheral Blood Mononuclear Cells (PBMC)

Heparinized peripheral blood was diluted 1 : 2 (vol/vol) in phosphate buffered saline (PBS), pH 7.2. PBMC were separated on a Ficoll density gradient by centrifugation at 1700 rpm for 30 min at room temperature (25 ± 3°C). After centrifugation, the interface cells were collected, washed twice, and counted using a hemocytometer to assess viability by trypan blue dye exclusion.

### 2.4. Isolation of CD4^+^ T Cells

CD4^+^ T cells were purified from PBMC by negative magnetic separation in a MiniMACS system. For all cell isolations, the manufacturer's instructions were followed. The purity of the separated CD4^+^ T cells was determined to be >95% in analyses through a flow cytometry (Becton & Dickinson FACScan; Mountain View, CA, USA), using an antihuman CD4 mAb coupled to FITC. Cell viability (>90%) of the purified cell subset was determined by the trypan blue dye exclusion method.

### 2.5. Inhibition Assays

Purified CD4^+^ cells were incubated with optimal concentration of biotin-labeled *ALL* in PBS supplemented with 0.2% bovine serum albumin and 0.2% sodium azide (PBA). After incubation, the cells were washed in PBA and incubated for a second step with PE-labeled streptavidin in PBA. To evaluate specificity of *ALL*, cells were again washed and incubated for the third time with 200 mM GalNAc in PBA. All incubations were carried out during 30 min at 4°C. Cells incubated only with PE-streptavidin were used as staining control.

### 2.6. Cell Cultures

CD4^+^ T cells were cultured in 24-well flat bottomed cell culture plates (Costar, Cambridge, MA, USA) at 1 × 10^6^ cells/well in RPMI-1640 medium supplemented with 1 mM sodium pyruvate, 2 mM L-glutamine, 50 *μ*g/mL gentamicin, and 10% heat-inactivated fetal calf serum and incubated at 37°C in a 5% CO_2_ humidified chamber. Concanavalin A (2 *μ*g/mL) was added and, after 48 h, the cells were harvested and processed to measure Foxp3, TGF-*β*, and CTLA-4 expression through flow cytometry. 

### 2.7. Immunofluorescence Staining of Cell Surface Markers

Triple-color staining was performed on purified CD4^+^ T cells by direct immunofluorescence, using FITC- or PE-labeled mAbs against CD45RA, CD45RO, CCR7, or CD25, and indirect fluorescence, using an optimal concentration of biotin-*ALL* plus CyChrome-labeled streptavidin. Briefly, 2 × 10^5^ cells were suspended in 20 *μ*L PBA and incubated with the fluorochrome-labeled mAb and biotin-*ALL* for 30 min at 4°C. After incubation, the cells were washed twice with PBA and incubated with CyChrome-streptavidin for 30 min. Then, cells were washed twice with PBA, fixed with 1% *p*-formaldehyde, and analyzed by flow cytometry.

### 2.8. Flow Cytometric Analysis of Intracellular Proteins

Purified CD4^+^ cells were incubated with biotin-*ALL* and CyChrome-streptavidin, as described above. Then, cells were fixed and permeabilized with the BD Cytofix/Cytoperm kit. Individually, cells were incubated with mAbs anti-IFN-*γ* FITC/IL-4 PE, anti-IL-10 PE, anti-Foxp3-FITC, or anti-CTLA-4-PE or anti-hLAP (TGF-*β*). After 30 min, cells were washed with PBS, incubated with anti-IgG-FITC for 30 min, and finally analyzed by flow cytometry. 

### 2.9. Flow Cytometric Analysis

All cells were analyzed for marker's expression by collecting 5000 events using a FACScan flow cytometer (Becton Dickinson, Mountain, View, CA, USA) and Cell Quest Pro software. To evaluate cell surface marker staining, a gate was drawn around the lymphocyte populations based on their physical properties (forward and side scatter), and a second gate was drawn based on positive or negative fluorescent *ALL*-binding to CD4^+^ T cells. To analyze intracellular protein staining, positive fluorescence staining of IFN-*γ*, IL-4, IL-10, TGF-*β*, Foxp3, and CTLA-4 (forward scatter and fluorescence) was set manually based on the distribution of cells stained with the isotype controls. Data are presented as two-dimensional dot plots or histograms. Control stains were performed using isotype-matched mAbs of unrelated specificity that were labeled with FITC or PE. Background staining was <1% and was subtracted from experimental values.

### 2.10. Statistical Analysis

Experiments were done independently and repeated at least three times. Data were analyzed by Mann-Whitney *U* test to detect significant differences. Analyses were performed with Sigma-Stat 3.1 software. Differences were considered statistically significant when *P* < 0.05. 

## 3. Results

### 3.1. Flow Cytometric Phenotypic Analysis

**Figure 1:**
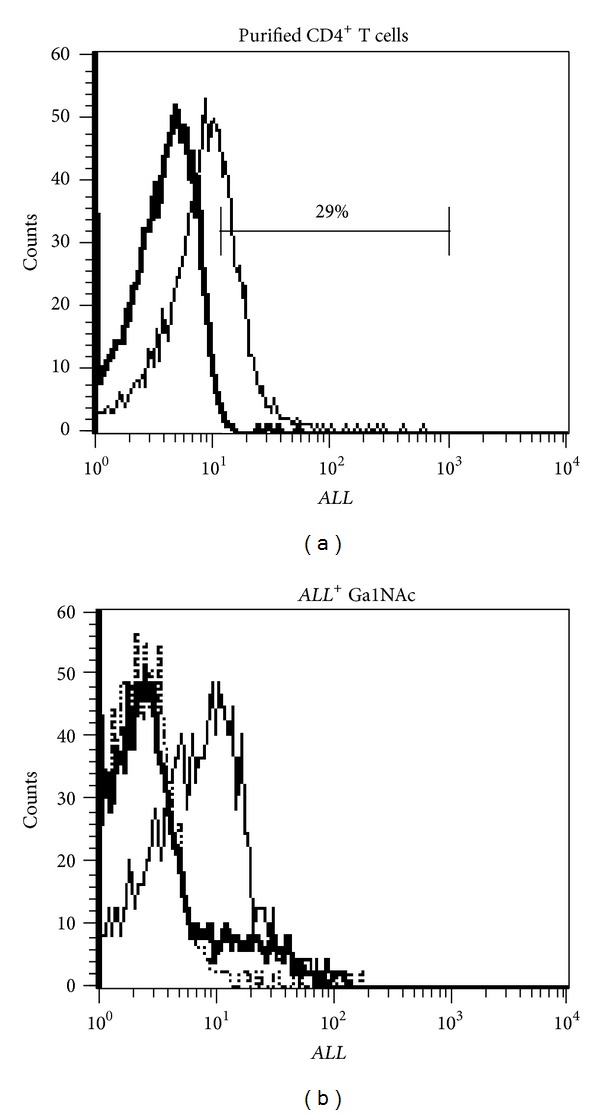
*ALL* recognizes purified CD4^+^ T cells. (a) Freshly purified CD4^+^ T cells were stained with CyChrome-labeled streptavidin alone (thick line) after incubation with biotin-labeled *ALL* (thin line). The bar denotes percentage of purified *ALL* ^+^CD4^+^ T cells. (b) GalNAc inhibited *Amaranthus leucocarpus* lectin (*ALL*) binding sites for CD4^+^T cells. Gray lines denote the staining of cells treated only with CyChrome-streptavidin; thin line indicates the florescence level of *ALL* ^+^ cells; thick line denotes fluorescence of *ALL* ^+^ cells when they were treated with GalNAc.

**Figure 2:**
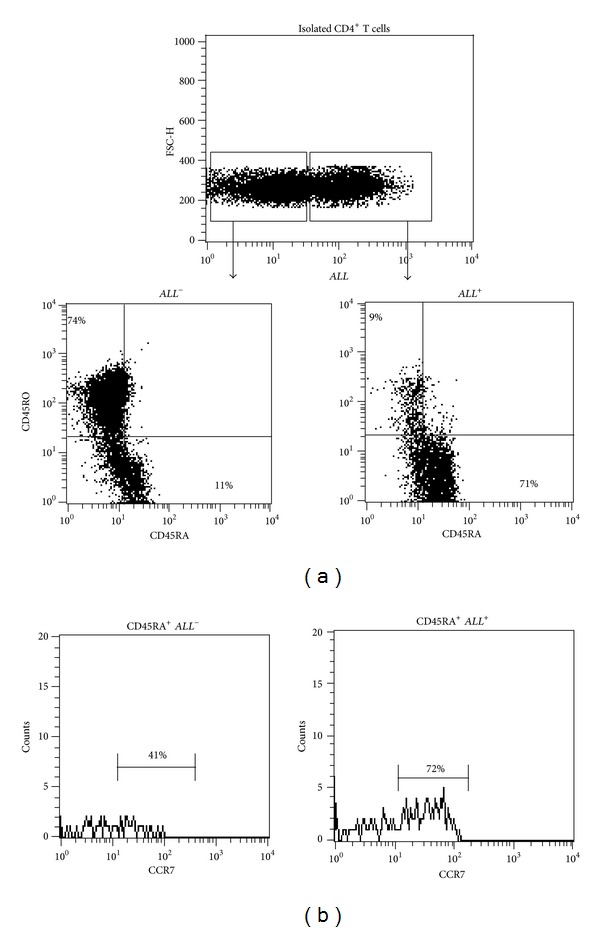
*ALL* recognition of CD4^+^ T cells with phenotype positive to CD45RA and CCR7 markers. (a) Dot plots of *ALL* ^−^ and *ALL* ^+^ gated cells showing frequency of CD45RO^+^ and CD45RA^+^ cells. (b) Representative histograms of CD45RA^+^ gated cells to analyze CCR7 frequency in both *ALL* ^−^ and *ALL* ^+^ T helper cells. The numbers denote percentage of cells positive to the marker.

The *ALL* ^+^ T cell subset corresponded to 29% of the purified CD4^+^ T cell population (Figure 1(a)). The specific interaction of *ALL* with a subset of CD4^+^ T cells was confirmed by inhibition assays with their characteristic ligand, as expected GalNAc inhibited most of *ALL*-binding sites for CD4^+^ cells (Figure 1(b)). Phenotypic characterization of both *ALL* ^+^ and *ALL* ^−^CD4^+^ T cells showed that the frequency of CD62L^+^ cells was 1.1 times higher in *ALL* ^+^CD4^+^ T cells (78% ± 1.4) than in *ALL* ^−^CD4^+^ T cells (69.2% ± 2.4) (*P* = 0.03). In *ALL* ^+^CD4^+^ T cells, the percentage of CD45RA^+^ cells was higher (71%) than the percentage of CD45RO^+^ cells (9%) (*P* = 0.02) (Figure 2(a)). We observed also that the frequency of CCR7^+^ cells was increased 1.5 times more in *ALL* ^+^CD4^+^ T cells (72%) than in *ALL* ^−^CD4^+^ T cells (41%) (Figure 2(b)).

### 3.2. Frequency of CD25 and Foxp3 on CD4^+^ T Cells

**Figure 3:**
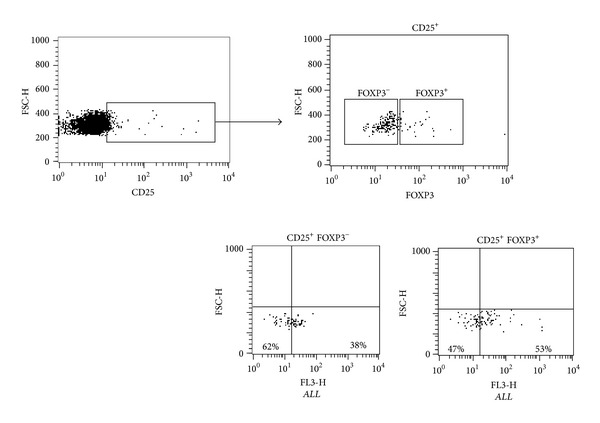
CD25 and Foxp3 frequency on *ALL* ^−^ and *ALL* ^+^CD4^+^ T cells. To analyze cell surface marker staining, a gate was drawn around the lymphocyte population based on their physical characteristics (forward) and positive fluorescence to CD25; a second gate was drawn based on positive or negative fluorescence to Foxp3. CD25^+^ cells positive or negative to Foxp3 were analyzed by binding to *ALL*.

Despite that the frequency of CD25^+^Foxp3^+^ cells was similar in both *ALL* ^+^ and *ALL* ^−^ T cells (Table 1), we observed that CD25^+^Foxp3^−^ cells were 1.9 times more frequent in *ALL* ^−^ T helper populations (62%) than in *ALL* ^+^ T helper cells (38%) (*P* = 0.001). Similarly, CD25^+^Foxp3^+^ cells were 1.3 times less frequent in *ALL* ^−^CD4^+^ T cells (47%) (*P* = 0.04) than in *ALL* ^+^CD4^+^ T cells (53%) (Figure 3).

### 3.3. Intracellular Cytokines in *ALL* ^+^CD4^+^ T Cells

**Figure 4:**
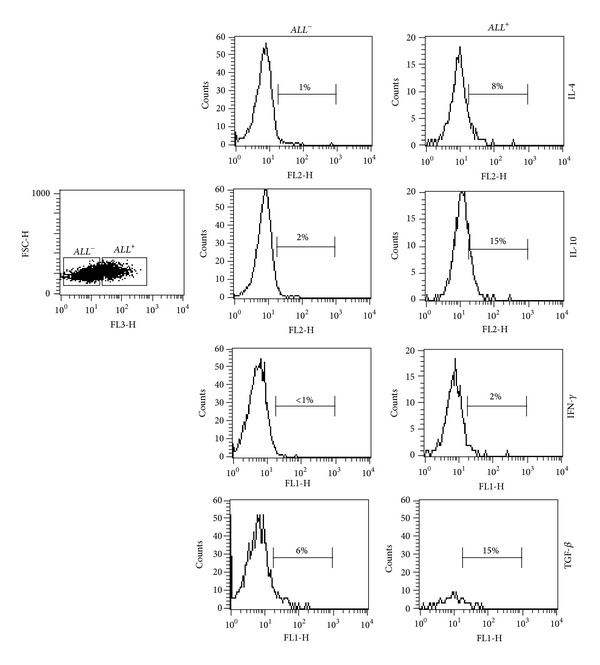
Frequency of intracellular cytokine positive cells on *ALL* ^−^ and *ALL* ^+^ gated cells. Freshly purified CD4^+^ T cells were incubated with *ALL*-Biotin followed by CyChrome-streptavidin incubation and stained with monoclonal antibodies against IL-4, IL-10, IFN-*γ*, or TGF-*β*. Cells were analyzed by flow cytometry and gated on *ALL* ^−^ or *ALL* ^+^ cells. Percentages of cells positive to intracellular cytokines are shown in histograms.

To know the cytokine profile of *ALL* ^+^ and *ALL* ^−^ T helper cells at basal conditions, we performed intracellular staining to the cytokines IL-4, IL-10, IFN-*γ*, and TGF-*β* in nonstimulated CD4^+^ cells. Our results showed that *ALL* ^+^CD4^+^ cells were IL-10^+^ (15%), IL-4^+^ (8%), IFN-*γ* ^+^ (2%), and TGF-*β* ^+^ (15%); in contrast, the percentages of cells positive to intracellular cytokines in the *ALL* ^−^CD4^+^ T cells subset were as follows: IL-10^+^ cells (2%), IL-4^+^ cells (1%), IFN-*γ* ^+^ cells (<1%), and TGF-*β* ^+^ cells (6%) (Figure 4).

### 3.4. Frequency of CD25, Foxp3, CTLA-4, and TGF-*β* Cells after Polyclonal Stimulation

**Figure 5:**
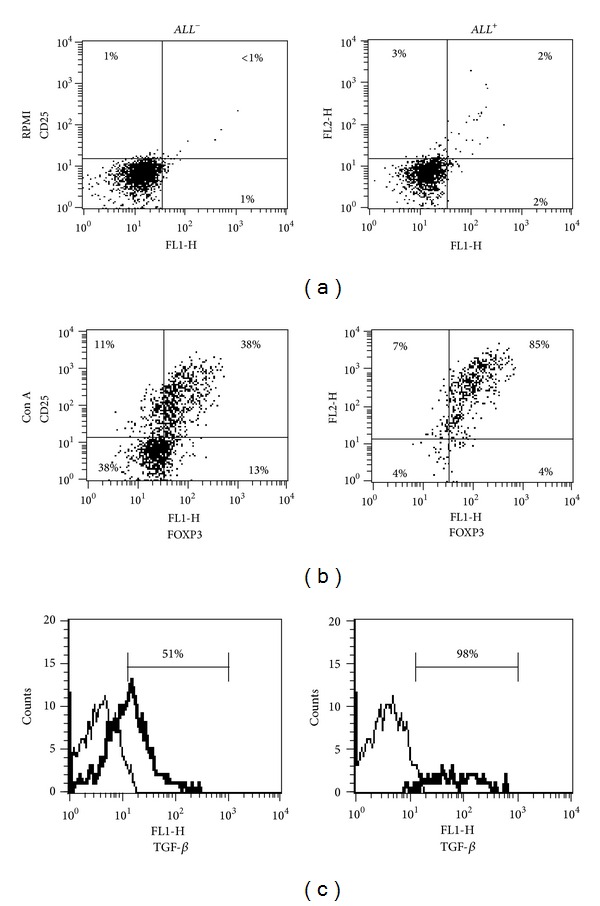
Polyclonal stimulation induced high expression of Foxp3 and TGF-*β* in *ALL* ^+^ T helper cells. CD4^+^ T cells were stimulated with Concanavalin-A (2 *μ*g/mL). After 48 h, cells were stained with biotin-*ALL* followed by CyChrome-streptavidin, and stained with monoclonal antibodies against CD25 and Foxp3 or TGF-*β*. (a) Dot plots of nonstimulated cells (RPMI alone) showing frequency of CD25^+^Foxp3^+^ in *ALL* ^−^ and *ALL* ^+^ T helper cells. After polyclonal stimulation An increased frequency of both (b) CD25^+^Foxp3^+^ cells and (c) TGF-*β* ^+^ cells (thick line) in *ALL* ^+^ T helper cells was observed. (Thin line corresponds to isotype control).

To determine whether the polyclonal stimulation influenced the frequency of CD25, Foxp3, and TGF-*β* in *ALL* ^+^ and *ALL* ^−^ cells, we performed an *in vitro* Con A stimulation assay during 48 hours. We observed that the frequency of *ALL* ^−^CD25^+^Foxp3^+^CD4^+^ T cells was increased 66.7 times in Con A-stimulated cells when they were compared to nonstimulated cells (*P* = 0.007), whereas the frequency of *ALL* ^+^CD25^+^Foxp3^+^CD4^+^ T cells was increased 2-times in ConA-stimulated cells as compared to nonstimulated cells (*P* = 0.036) (Figure 5(a)). Despite the increment in the number of CD25^+^Foxp3^+^CD4^+^ T cells in the *ALL* ^−^ cell subset, the percentage of CD25^+^Foxp3^+^CD4^+^ predominated in the *ALL* ^+^ cell subset (*P* = 0.007) at the end of the culture (Figure 5(b)). Although, we did not find statistical differences in intracellular/surface frequency of CTLA-4^+^ cells among *ALL* ^+^ (37.5 ± 6.5) and *ALL* ^−^ (15.1 ± 2) T helper cells (*P* = 0.05) (data not shown); the percentage of TGF-*β* ^+^ cells was statistically higher in *ALL* ^+^CD4^+^ T cells (98%) than in *ALL* ^−^CD4^+^ T cells (51%) (*P* = 0.02) (Figure 5(c)). After polyclonal stimulation, the percentage of TGF-*β* ^+^ T cells increased 2.6 times in the *ALL* ^+^CD4^+^ T cell subset as compared to *ALL* ^−^CD4^+^ T cells; no significant differences were observed when we compared Con A-stimulated *ALL* ^−^CD4^+^ T cells with nonstimulated cells (Table 2).

## 4. Discussion

Surface O-glycosylation pattern of lymphocytes has been involved in development, maturation, homing, and immune regulation [24, 25, 29]. It has been shown that glycosylation changes occur in activated lymphocytes [26], and differences in sialylation as well as in expression of O-glycans are related to control of T-cell activation [30]. In this study, we found that a subpopulation of CD25^+^Foxp3^+^CD4^+^ T cells expressing galactose-N-acetylgalactosamine or N-acetylgalactosamine is recognized by the *Amaranthus leucocarpus* lectin. A significant percentage of freshly obtained *ALL*-recognized T cells exhibited intracellular cytokines with regulatory activity such as IL-10 and TGF-*β*. It has been shown that CD25^+^CD4^+^ Treg cells are able to produce IL-4 and IL-10, without production of IFN-*γ* [23]. Likewise, other authors have shown that sorted CD25^+^CD4^+^ Treg cells produce high concentrations of IL-10 and low IFN-*γ* [22]. In agreement with these data, our results suggest that *ALL* ^+^ T cells correspond to a Treg cell subset.

Previous reports concerning *ALL* binding to T cells suggest that this lectin recognizes specific O-glycans on recently activated naïve T cells [31]. Our results showed that ~29% of the purified CD4^+^ T cells were positive to *ALL*. Phenotypic characterization of *ALL* ^+^CD4^+^ T cells included a subset of CD45RA^+^, CCR7^+^, CD25^+^, and Foxp3^+^ cells, which resemble natural naïve NnTregs [32]. The NnTregs represent a major lymphocyte population engaged in the control of self-reactive T responses and in maintenance of immune tolerance [18, 20]. Along with our findings, CD4^+^ T cells recognized by *ALL* were reported CD45RA^+^CD27^+^ cells [12]. The CD27 molecule is a member of the TNF receptors that have been involved in early activation of naïve cells [33]. It is interesting to note that CD27 expression on CD4^+^ T cell lines discriminates between regulatory and nonregulatory cells [33, 34]. Different authors have reported a distinct subset of CD25^+^Foxp3^+^CD4^+^ T cells characterized by CCR7, CD62L, and CTLA-4 expression contained in the CD45RA^+^/RO^−^ naïve compartment [22, 23, 35]. CCR7 is a chemokine receptor that controls homing of lymphocytes to secondary lymphoid organs, and its expression in Tregs has been associated with maintenance of these cells for prolonged periods of time at those sites, inhibiting effector T-cell expansion [21]. CD62L and CCR7 expression in T cells mediates lymphocyte homing to secondary lymphoid organs [36]. CTLA-4 is a cell surface molecule that is expressed rapidly before cell activation, surface CTLA-4 is immediately internalized, which could explain the low levels of expression generally detected on the cell surface [37]. Diverse authors have related CTLA-4 expression with nTreg cells [22, 23], and CTLA-4 expression is increased in Treg > NnTreg. Consistent with these data, we observed that the percentage of CTLA-4^+^ cells was increased in *ALL* ^+^ than in *ALL* ^−^CD4^+^ T cells, suggesting that CD4^+^ cells recognized by *ALL* could be either a subpopulation enriched in NnTreg cells or recently activated Treg cells [23]. A proposal could be explained based on results obtained with *in vivo* antigenic-experienced circulating cells, as has been reported for other proteins related to Treg cells, such as Foxp3 [19], or for the intracellular CTLA-4 [22, 23].

Regulatory T cells are resistant to apoptosis [38]; similarly, *ALL* ^+^ thymocytes are resistant to apoptosis after treatment with dexamethasone [14]; for this reason it would be interesting to examine apoptosis resistance in the *ALL* ^+^CD4^+^ T cells from human peripheral blood to understand better the potential regulatory characteristics of this *ALL* ^+^ NnTreg cell subset. Golks et al. [26] showed that an N-acetylglucosaminyltransferase is required for T cell activation, suggesting that modifications in glycosylation accompany T cell activation as reviewed [29]. In this regard, the glycosylation status could generate differences among subsets of T cells, and possibly Tregs, as we observed in this study.

Taken together, our results suggest that *ALL* constitutes an important tool to study differences in O-glycans on CD4^+^ T cells with a regulatory-like phenotype, these CD4^+^ T cells are enriched NnTregs or recently activated Tregs expressing galactose-N-acetylgalactosamine and N-acetylgalactosamine. 

## Figures and Tables

**Table 1:** Recognition of CD25^+^Foxp3^+^CD4^+^ T-cell subsets by* Amaranthus leucocarpus* lectin (*ALL*).

CD4^+^T cells			
CD25^+^Foxp3^−^ cells		CD25^+^Foxp3^+^ cells	
*ALL* ^ +^	*ALL* ^−^	*ALL* ^ +^	*ALL* ^−^
34 ± 4*	65 ± 4^∗‡^	52 ± 10	50 ± 7^‡^

*P = 0.001, ^‡^ *P* = 0.004.

**Table 2:** Frequency of TGF-*β* ^+^ cells on purified CD4^+^ cells.

Con A-stimulated	Nonstimulated
*ALL* ^ +^ cells 94 ± 6*	4 ± 2*
*ALL* ^−^ cells 38 ± 18	6.7 ± 5.7

*P = 0.003.
